# Bacteriophage resistance evolution in a honey bee pathogen

**DOI:** 10.1099/mic.0.001595

**Published:** 2025-08-20

**Authors:** Emma K. Spencer, Yva Eline, Lauren Saucedo, Kevin Linzan, Keera Paull, Craig R. Miller, Tracey L. Peters, James T. Van Leuven

**Affiliations:** 1 Department of Biological Sciences, University of Idaho, Moscow, ID, USA; 2 Institute for Modeling Collaboration and Innovation, University of Idaho, Moscow, ID, USA; 3 National Summer Undergraduate Research Project, UC Davis, California, USA; 4 Department of Animal Veterinary and Food Sciences, University of Idaho, Moscow, ID, USA

**Keywords:** bacteriophage therapy, bacteriophage resistance, honey bees, *Paenibacillus larvae*

## Abstract

Honey bee (*Apis mellifera*) larvae are susceptible to the bacterial pathogen *Paenibacillus larvae*, which causes severe damage to bee colonies. Antibiotic treatment requires veterinary supervision in the USA, is not used in many parts of the world, perpetuates problems associated with antibiotic resistance and may necessitate residual testing in bee products. There is interest in using bacteriophages to treat infected colonies (bacteriophage therapy), and several trials are promising. Nevertheless, the safety of using biological agents in the environment must be scrutinized. In this study, we analysed the ability of *P. larvae* to evolve resistance to several different bacteriophages. We found that bacteriophage resistance rapidly developed in culture but often results in growth defects. Mutations in the bacteriophage-resistant isolates are concentrated in genes encoding potential surface receptors but are also observed in genes controlling general cellular functions and in two cases – lysogeny. Testing one of these isolates in bee larvae, we found it to have reduced virulence compared to the parental *P. larvae* strain. We also found that bacteriophages are likely able to counteract resistance evolution. This work suggests that while bacteriophage resistance may arise, its impact will likely be mitigated by reduced pathogenicity and secondary bacteriophage mutations that overcome resistance.

## Data Availability

Analysis scripts and processed data were published on Github at https://github.com/jtvanleuven/plarvae_resistance. Supplementary material is available with the online version of this article, available through Figshare at https://doi.org/10.6084/m9.figshare.29098688 [1]. Genome assemblies and raw sequencing reads for the seven starting phages (SRR31700794-SRR31700800) and the ancestor (starting isolate) of B-3650 (SRR31700744, SRR31700745) were deposited at National Center for Biotechnology Information (NCBI) under BioProject number PRJNA1130131. Raw sequencing reads (SRR29659251-SRR29659276) for the evolved phage-resistant isolates of B-3650 were deposited under the same BioProject number.

## Introduction

Managed honey bees (mainly *Apis mellifera*) pollinate around one-third of the world’s pollinator-dependent crops, making their health critical to our food security and integral to food prices. In total, managed honey bees provide $182–577 billion USD/year in global crop pollination services [2,3]. Unfortunately, beekeepers regularly lose around one-third of their colonies every year to a combination of disease and other stressors [4,5]. Efforts to prevent these losses and replace colonies are costly. Among the diseases that contribute to the operational costs of beekeepers is American Foulbrood (AFB). AFB is caused by the Gram-positive, spore-forming bacteria *Paenibacillus larvae*. This disease stands out as particularly devastating because of its limited treatment options. In the USA, veterinarian-prescribed antibiotics can be used to clear *P. larvae* infections, but the emergence of antibiotic-resistant strains of *P. larvae* raises concerns over AFB management [6,9]. Moreover, antibiotics may not eliminate * P. larvae* spores, which can remain in the hive for decades [10,12]. In many European Union countries, regulations over antibiotic use and the level of antibiotic residues in bee products curtail their use. As a response to this crisis, researchers have begun to investigate the use of bacteriophages – viruses that specifically target and infect bacteria – as potential allies in the fight against AFB [13,19]. These bacterial predators hold immense promise due to their precision, efficacy and eco-friendly nature [20,21]. Bacteriophages (phages) present an attractive solution to the problem of antibiotic resistance.

**Fig. 1.**
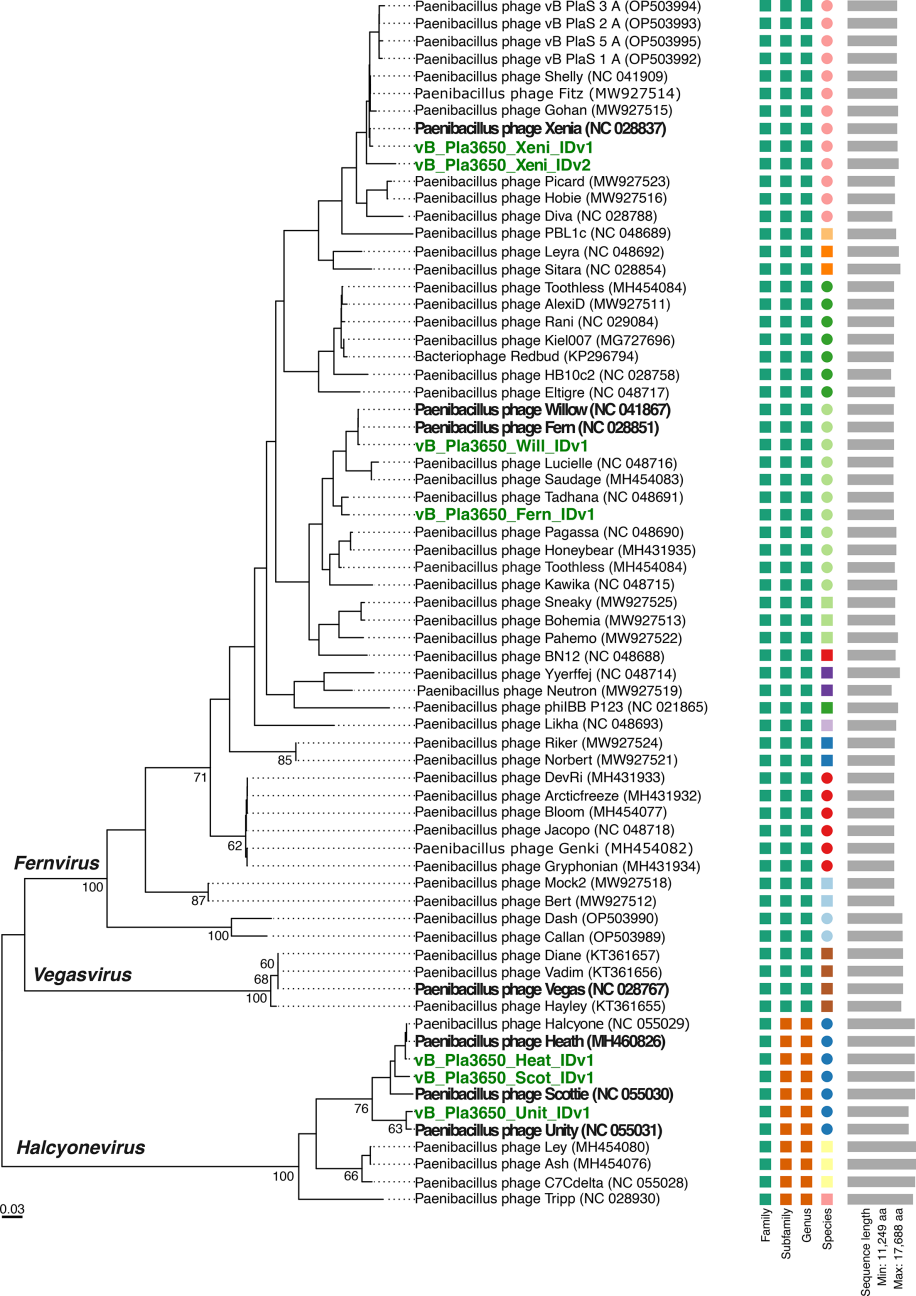
Phylogenomic Genome BLAST Distance Phylogeny (GBDP) tree inferred using the D6 formula based on whole-genome amino acid sequence pairwise comparisons, with average support of 31%. The numbers above the branches are the pseudo-bootstrap values from 100 replications, using the VICTOR workflow. International Committee on Taxonomy of Viruses (ICTV) genus designations are listed on respective branches (*Fernvirus*, *Vegasvirus* and *Halcyonevirus*). Phages used in this study are emphasized with green bold text. Parent phages that they are derived from are emphasized in black bold text.

Many phages that infect *P. larvae* have been isolated, and attempts have been made to classify them according to the ever-changing universal viral taxonomy (https://ictv.global/about/charge) to the genus and species level, such as those phages included in the genera *Fernvirus*, *Vegasvirus* and *Halcyonevirus* (Fig. 1) [13,14, 16,18, 22]. These phages can kill nearly all known *P. larvae* genotypes in culture [13,22] and reduce disease burden when tested on bee hives [14,18]. However, all discovered *P. larvae* phages are temperate, a potentially problematic property for their use as therapeutics because of lysogeny [26] and the ability of temperate phages to transfer genetic information between hosts by generalized and specialized transduction [27,28]. The host range (breadth of susceptible host genotypes) of *P. larvae* phages is very broad – a positive characteristic for their utilization in treating infected hives. Thus, phage cocktails comprised of only a few phages can be designed to treat all or nearly all *P. larvae* genotypes that currently infect *A. mellifera*. The genetic diversity of *P. larvae* genotypes is organized by clustering variants into five subgroups based on enterobacterial repetitive intergenic consensus (ERIC) amplification patterns [29,30]. These subgroups differ in their geographic distribution, prevalence and infection characteristics. ERIC I and II are currently the most widespread and problematic [31,33].

In this study, we assessed the evolutionary capacity of an ERIC I *P. larvae* isolate NRRL B-3650 to gain resistance to phages and identified the genetic determinants of this phage resistance. In addition, we assessed how these genetic mutations affected host growth rate and analysed how the acquisition of resistance to one phage affects an isolate’s susceptibility to other phages. This study will help us design effective phage cocktails and understand potential problems when using phage therapy to treat infected animals.

## Methods

### Bacterial and phage strains and culture conditions

*P. larvae* strain NRRL B-3650 and all seven phages were obtained from Dr. Penny Amy (University of Nevada, Las Vegas). Glycerol stocks of B-3650 were streaked on brain heart infusion (BHI) agar plates for the isolation of a single genotype (2X re-streaks). A single colony was used to inoculate 3 ml of BHI liquid media, which was subsequently shaken at 200 r.p.m. at 37 °C in 5% CO2 for ~30 h to an OD_600_ of ~0.7. Glycerol stocks of the seven phages were revived by mixing freezer stock with 100 µl of OD_600_ ~0.7 cells (~1x10^7^ c.f.u. ml^−1^) in warm modified brain heart infusion (mBHI) top agar (BHI, 1 mM CaCl2, 1 mM MgCl2 and 0.7% agar). The warm top agar containing phage and bacteria is gently poured onto a solid agar plate and allowed to dry for ~30 min at room temperature, at which point they are moved to a 37 °C, 5% CO2 incubator for overnight growth. A single plaque was picked and amplified to high titre overnight in 3 ml of mBHI with B-3650. New phage stocks were generated by pelleting cellular debris from spent cultures (1,000 ***g***) then chloroform treating the supernatant (50 µl of chloroform added to 500 µl supernatant). Residual chloroform was removed by centrifuging for 4 min at 13,000 ***g*** and pipetting off the supernatant containing phages. These stocks were titered and stored at 4 °C for immediate use.

### Isolation and characterization of phage-resistant mutants

**Fig. 2.**
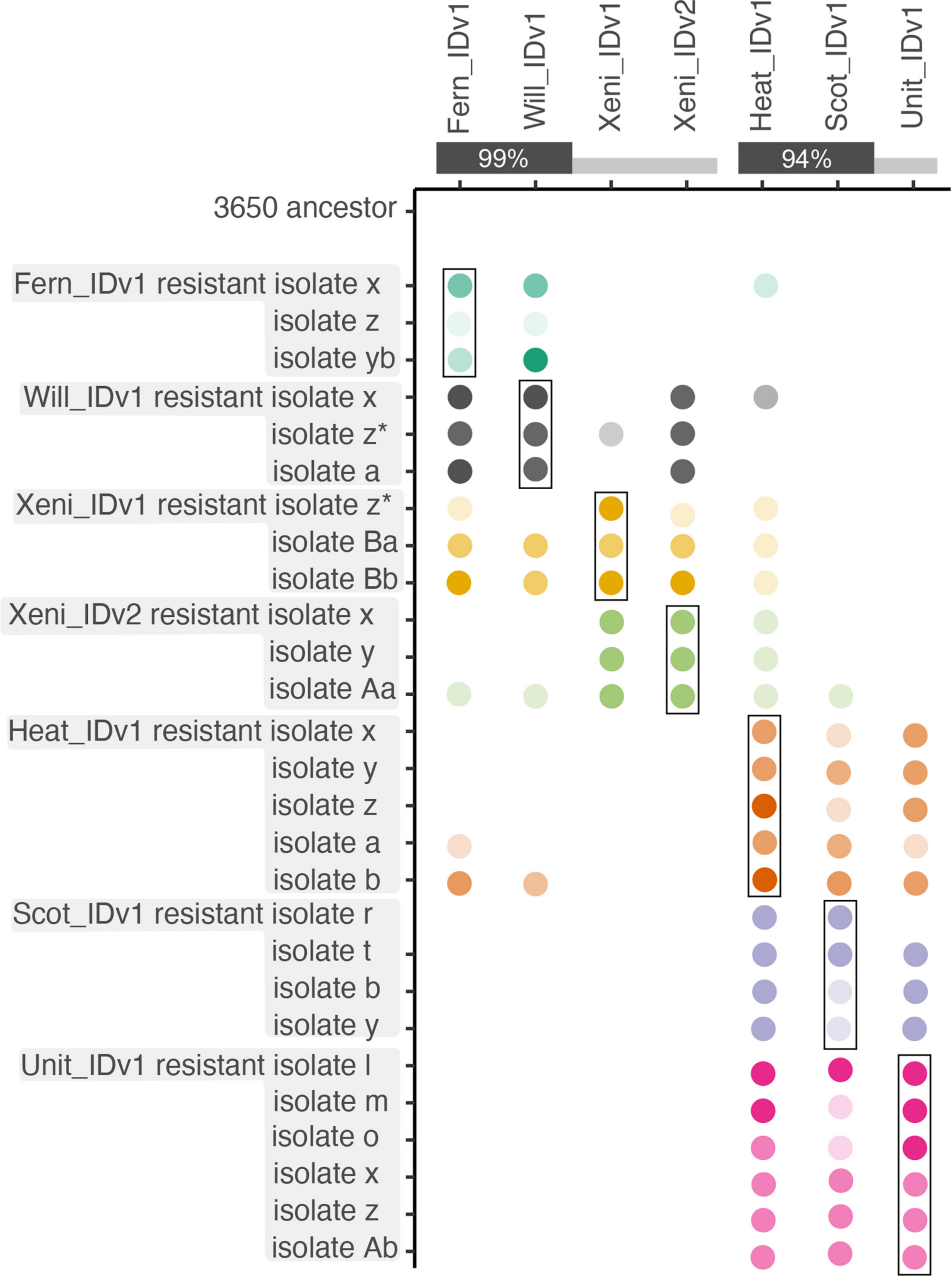
Phage resistance evolution protects hosts against closely related phages. We tested the susceptibility of all phage-resistant isolates (*y*-axis) to seven different phages (*x*-axis). The amount of bacterial growth in the presence of the phages listed along the *x*-axis is shown by the opacity of coloured circles. Dark circles indicate complete or near-complete resistance. Partial resistance is indicated by opaque circles. The absence of a circle means that the isolate was killed (no resistance) by the phage named along the top (*x*-axis) label. The boxes indicate the results of testing a host isolate against the phage that was used to evolve resistance. Nucleotide sequence identity between phages is shown along the top. * indicates subtle differences in host range among the three methods used. These methods included cross-streaking on agar plates, spot plating phage lysates on bacterial lawns and soft-agar overlay (see ‘Methods’ for details).

For the resistance evolution experiment, we streaked out a loop-full of glycerol stock B-3650 on BHI agar plates. A colony was chosen for each replicate and grown in 3 ml BHI to an OD6_00_ of ~0.7. Three random marks were placed on the bottom of the agar plate, and then 100 µl of cells were briefly vortexed with phage stocks (litres provided in Table S2, avaiable in the online Supplementary Material) at a multiplicity of infection (MOI) of 5 in mBHI top agar (0.7% agar) and gently poured on solid BHI agar plates. Plates were incubated for 72 h at 37 °C in 5% CO_2_. Cleared plates with phage-resistant colonies were visible between 24 and 72 h, and the three colonies closest to the marks were picked. This was repeated at least twice (two starting colonies) for each challenge phage except for Unit_IDv1, which required a third replicate to acquire sufficient plaques. In total, four colonies were used to spread the evolution experiments across more days. Table 1 shows which of the four parental colonies each phage-resistant isolate originated from. After 72 h, the following colony counts were observed: four colonies for Heat_IDv1, 7 colonies for Scot_IDv1, 9 colonies for Unit_IDv1 and 114 colonies for Xeni_IDv1. To confirm resistance, individual colonies were picked, grown overnight in mBHI broth and streaked across phage stock (phage streak assay) that was dibbled perpendicular to the colony streak (for example, see Fig. S3). Volumes of 20–50 µl of phage stock were dripped across the agar plate and allowed to soak into the agar for ~2 min. A 10 µl loop-full of overnight culture from putatively resistant hosts was streaked perpendicular to the phage. The B-3650 ancestor was always included as a control. This same phage streak assay was used to determine cross-resistance shown in Fig. 2. Two additional tests were used for cross-resistance testing. First, we spot plated 5 µl of phage stock on lawns of B-3650 and its derivative phage-resistant isolates. Phage stock litres for the spot plating are shown in Table S2. Lastly, phage stocks were titered on every host using the top agar method, as mentioned earlier. The litres were then compared to B-3650 to calculate the efficiency of plating (EOP). A linear model where EOP is dependent on the type of host (resistant host evolved on challenge phage versus resistant host with susceptibility to other phages) was compared to a null model with ANOVA (*P*-value comparing models=9.1E−5); then, a post hoc Tukey HSD test was performed. The full matrix of litres (including survivor phage isolates) on every host isolate is provided in Table S2.

**Table 1.** Comparison between *Paenibacillus* phages used in this study (designated by an asterisk and a single letter, e.g. Fern_IDv1=F) and previously published reference phage genomes (designated by outlined cells and bold text) using whole-genome average nucleotide identity† Lifestyle prediction is shown. Phages were propagated on *P. larvae* sbsp. *larvae* strain B-3650.

*See references 9 and 17 for clade details.

†As determined by fastANI, adjusted for number of fragments aligned, reciprocal ANI scores averaged and comparisons too low for fastANI to calculate are shown as ‘na’; see reference 72 and methods.

### Growth curves of phage-resistant isolates

The OD_600_ of cultures of 14 phage-resistant isolates of *P. larvae* (6 that are resistant to *Halcyoneviruses* and 8 resistant to *Fernviruses*) was measured in 24-well shaking plate assays in an incubating plate reader (BioTek Instrument, Inc., USA). Control wells included BHI broth control, bacteria control and phage control. The plates were incubated while shaking for 48 h with the reading of OD (600 nm) recorded from each well at an interval of every 15 min (Table S3). OD_600_ readings were analysed in R using the gcplyr (version 1.6.0) and lme4 (version 1.1–33) packages [34]. Using the gcplyr package, we extracted doubling time, maximum density and lag time from the growth data. We compared linear models for these three metrics that included the following predictor variables: phage type, isolate and mutated gene using Akaike information criterion (analysis code is available at https://github.com/jtvanleuven/plarvae_resistance). This analysis identified all three predictor variables as having a significant impact on at least one of the dependent variables. Thus, we performed three ad hoc linear models to identify which isolates were significantly different from B-3650.

### Sequencing and analysis

Overnight cultures of strain B-3650 and phage-resistant isolates were harvested by centrifugation and resuspension in lysis buffer followed by DNA isolation (ZymoBIOMICS). Phage whole-genomic DNA was extracted using the Norgen Phage DNA Extraction Kit (optional protease and second elution steps were included). Phage and host strain B-3650 gDNA were submitted for sequencing on an Illumina HiSeq platform (150PE) with Omega (Norcross, GA). B-3650 gDNA was also prepared for Nanopore sequencing using Native Barcoding Kit 24 V14 (SQK-NBD114.24) and sequenced on a MinION device. Bases were called using dorado (dna_r10.4.1_e8.2_400bps_sup@v4.2.0, https://github.com/nanoporetech/dorado) and demultiplexed using guppy (https://github.com/rrwick/Filtlong). Illumina sequencing of bacterial strain B-3650 as a control and 26 phage-resistant isolates was performed by SeqCoast Genomics (Portsmouth, NH). Samples were prepared for whole-genome sequencing using an Illumina DNA Prep Tagmentation Kit and unique dual indexes and sequenced on an Illumina NextSeq2000 platform using a 300-cycle flow cell kit (150PE).

Illumina reads were quality filtered and trimmed using fastp (v0.23.4). Nanopore reads were filtered using filtlong (v0.2.1) (https://github.com/rrwick/Filtlong). Unicycler (v0.5.0) was used to assemble phage genomes using the short-read approach with default parameters. Where necessary, phage samples were subsampled to achieve a depth of 100× prior to assembly using seqtk version 1.4-r122 to achieve complete assemblies. A hybrid assembly was produced using Unicycler [35] for strain B-3650 and was annotated using Bakta [36]. This assembly served as the reference genome for variant analysis. Our hybrid assembly of strain B-3650 was compared to strain NZ_CP019651 by pairwise alignment. One SNP at genome position 365,471 and 1 single-base indel at genome position 2,269,949 compared to CP019651 were identified. These mutations are in a putative transposase and mobile element protein, respectively. Variant analysis of phage-resistant isolates was conducted by mapping Illumina reads to the hybrid B-3650 reference genome using BREseq v0.38.1 and visualized using gggenomes [37]. Custom R scripts (https://github.com/jtvanleuven/plarvae_resistance) were used for data processing and plotting. Additional mapping was performed using bwa mem (0.7.18-r1243-dirty) and visualized in Tablet (v1.21.02.08).

Phage genomes were annotated using pharokka [38] and assembly statistics were generated using bbmap and samtools. Whole-genome based phylogeny of our seven phages and *Paenibacillus* phages of the genus *Fernvirus*, *Halcyonevirus* and *Vegasvirus* was conducted using the VICTOR pipeline [39]. Previously published phage genomes were first downloaded from National Center for Biotechnology Information and re-annotated using pharokka and then submitted to VICTOR whole-genome amino acid analysis. Whole-genome average nucleotide identity calculations were made using fastANI [40], which reports average nucleotide identity and the number of aligned fragments. Because some of the phages in our study are quite divergent but may have short regions of high nucleotide identity, we adjusted the average nucleotide identity (ANI) score by multiplying it by the fraction of the genome aligned. We also averaged the two ANI values that fastANI reports from its reciprocal mapping. This was done to make an easier-to-interpret triangular matrix that is reported in Table 1. fastANI does not report ANI values below around 75%; thus, cells in Table 1 containing NA are those for which fastANI did not report scores. ANI values in Table 1 that are below 75% are a result of our scaling by proportion of the genome matched (e.g. fastANI reported a score, but these nucleotide matches only spanned a portion of the phage genome). PhaMMseqs [41] was run on all phage GenBank files using default parameters, and the resulting phamilies were imported into SplitsTree 6 [42] after creating a matrix of phamilies in R. We did not expect to obtain two versions of the Vegas phage; thus, we carefully compared the sequence of vB_Pla3650_Vega_IDv2 to vB_Pla3650_Vega_IDv1, and the prophage regions in B-3650 to ensure that vB_Pla3650_Vega_IDv2 is unique and not an induced prophage. vB_Pla3650_Xeni_IDv1, vB_Pla3650_Xeni_IDv2, Xenia NC_028837.1, Fern KT361649 and prophage regions from *P. larvae* B-3650 all share syntenic genome regions consisting of multiple genes with nucleotide identities up to 99% [43]. However, all have unique gene content and have multiple genes that are quite diverged (Fig. S2). The analysis code is provided in https://github.com/jtvanleuven/plarvae_resistance.

### Virulence assay

Age-matched 1–3-day-old larvae were transported from our apiary to our laboratory in a pre-heated foam nuc box containing a bottle of hot water and were covered with a damp paper towel. Within 30–60 min from being removed from the hive, larvae were grafted using a Chinese grafting tool into brown queen cups in 48-well tissue culture plates containing 20 µl of preheated artificial food ‘A’ (44.25% royal jelly, 5.3% glucose, 5.3% fructose, 0.9% yeast extract and 44.25% water). Larvae were incubated for 48 h (see conditions below), and then 20 µl of artificial food ‘B’ (42.95% royal jelly, 6.4% glucose, 6.4% fructose, 1.3% yeast extract and 42.95% water) was added to the cells. Plates were photographed daily, and death was measured by observing larval discolouration and the absence of diet consumption. Larvae were reared following standard procedures [44]. Temperature and humidity were monitored constantly and remained at 35 °C and 90–100% humidity. One hundred c.f.u of either B-3650 or Fern_IDv1-resistant isolate yB vegetative cells in 1 µl of mBHI growth media were added to the artificial food on day zero. Mock-infected larvae had 1 µl of mBHI added to their food. Larval survival was compared in R using the ‘survival’ and ‘survminer’ packages. The Cox proportional hazards regression model was fit according to the following formula: (days post-infection, survival) ~ treatment. *P*-values were calculated for all pairwise comparisons using log-rank tests with Benjamini–Hochberg multiple test correction [45].

## Results

### Re-sequencing of host strain B-3650 and seven *Paenibacillus* phages

We first sought to establish a reliable reference genome for host strain B-3650. To accomplish this, we used both long- and short-read data to create a hybrid assembly. This approach resulted in an assembly with one complete contig of 4,355,922 bp that was then annotated using Bakta.

All seven phages used in this study were originally obtained from Dr. Penny Amy at the University of Nevada, Las Vegas, and were amplified for experimental use on host strain B-3650. To verify the genetic identity of these phages, we re-sequenced each phage genome used for selection and gave them new naming designations as follows: vB_Pla3650_Fern_IDv1, vB_Pla3650_Xeni_IDv1, vB_Pla3650_Xeni_IDv2, vB_Pla3650_Will_IDv1, vB_Pla3650_Heat_IDv1, vB_Pla3650_Scot_IDv1 and vB_Pla3650_Unit_IDv1. Phage reads and genomes were submitted to NCBI (see ‘Data availability’). We then compared sequence identities between these seven phages and published *Paenibacillus* phage reference genomes (Fig. 1, Table 1, Fig. S1, Table S1). Whole-genome average nucleotide alignments of phages to their respective reference genome showed sequence identities between 99.81% (Heat_IDv1 to Heat) and 89.89% (Fern_IDv1 to Fern). A phage isolate that we anticipated to be Vegas showed very low identity (29.53%) to the published Vegas genome. This phage was most closely related to phage Xenia (92.16%), and Mash distance estimation classified this phage as a *Fernvirus*. We subsequently named this isolate Xeni_IDv2. vB_Pla3650_Xeni_IDv2 is distinct from Vegas and from prophage regions in B-3650, which do share high nucleotide similarity across some genes (Fig. S2).

### Rapid resistance evolution

**Fig. 3.**
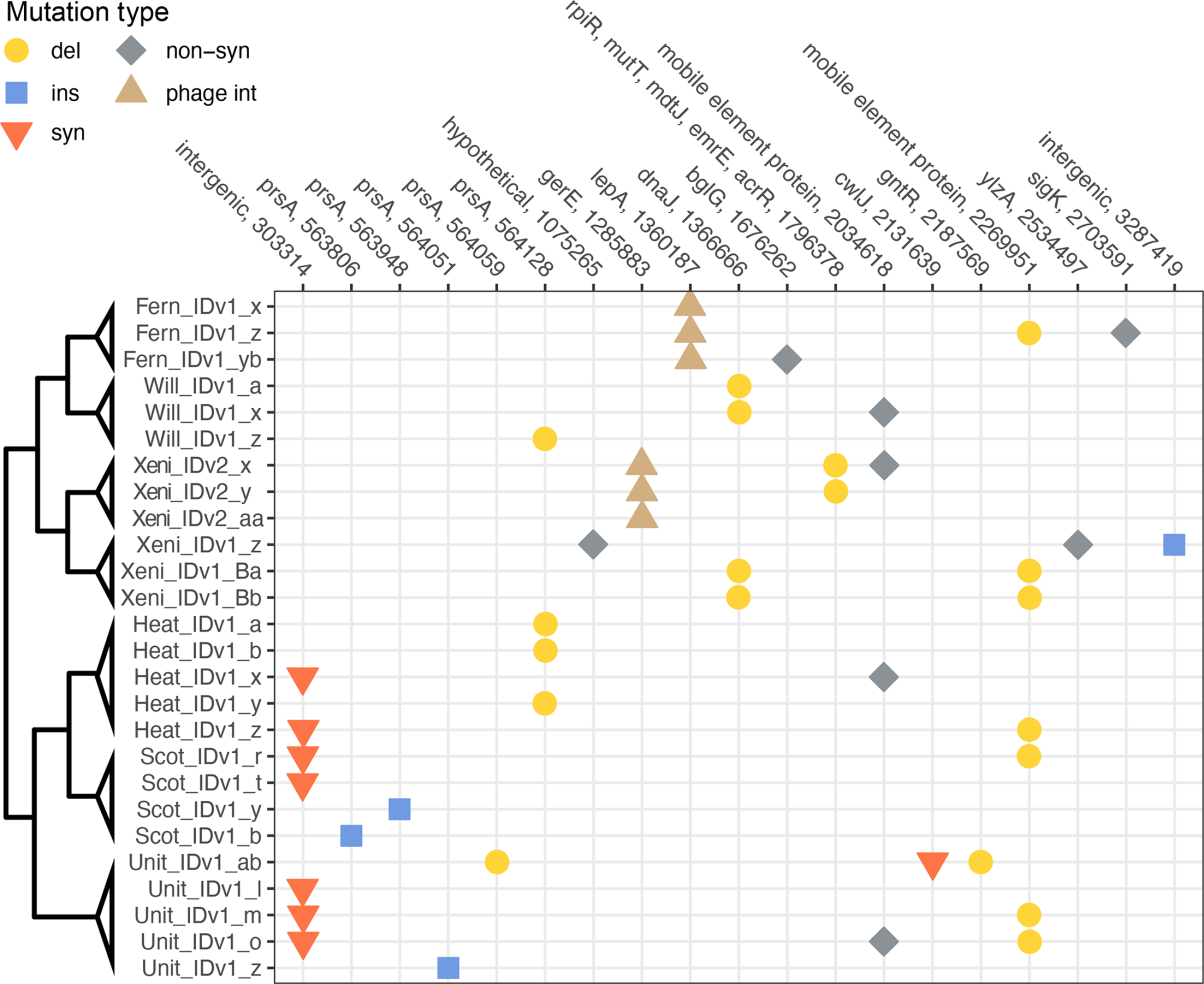
Heatmap depicting the location of mutations in phage-resistant *P. larvae* sbsp. *larvae* strain B-3650 isolates selected by each phage (Fern_IDv1, Heat_IDv1, Scot_IDv1, Unit_IDv1, Xeni_IDv2, Will_IDv1 and Xeni_IDv1). Individual isolates are denoted by unique letters following the phage name with which they were challenged. Mutation type is designated as follows: del, deletion (circle); ins, insertion (square); non-syn, non-synonymous mutation (diamond); phage int, formation of lysogen by phage integration (triangle); syn, synonymous mutation (upside-down triangle). Variant analysis was conducted using breseq. The cladogram identifies related groups – branch lengths do not show genetic distance.

To select for phage-resistant isolates, we challenged lawns of *P. larvae* strain B-3650 (ERIC I) with seven different phages (Table 1) at an MOI of around 5. After 24–72 h, phage-resistant colonies were visible on otherwise cleared plates in all seven challenges. We picked 3 to 5 colonies from each phage challenge, re-confirmed their resistance to the phage initially used and sequenced the genomes of 26 isolates. These isolates were named according to the phage that they were challenged with and a one- or two-letter suffix to denote individual isolates (e.g. Fern_IDv1-resistant isolate yb). In these 26 isolates, a total of 19 unique mutations (13 unique genes) were observed (Fig. 3, Table 2).

**Table 2.** Mutations identified in B-3650 phage-resistant isolates selected by seven *Paenibacillus* phages Superscripts (1–4) indicate different clones (colonies) from one agar plate that initiated the sub-cultures used to evolve resistance. SNP, ins, insertion; del, deletion; SV, structural variant; non-syn, non-synonymous; FS, frameshift.

Genomic position	**Gene name**	**Predicted function**	**Mutation type**	Nucleotide change	**Mutation effect**	Number of isolates selected by phage							Total isolates with mutation	Isolate ID
						*Fernvirus*				*Halcyonevirus*				
						Xeni IDv2	Xeni IDv1	Fern IDv1	Will IDv1	Scot IDv1	Heat IDv1	Unit IDv1		
303,314	Intergenic		SNP	G->A	Unknown					2	2	3	7	Heat_IDv1_x^1^, Heat_IDv1_z^1^, Scot_IDv1_r^2^, Scot_IDv1_t^2^, Unit_IDv1_l^3^, Unit_IDv1_m^3^, Unit_IDv1_o^3^
563,806	*prsA*	Foldase	SV		Disruption of the coding region					1				Scot_IDv1_b^4^
563,948	*prsA*	Foldase	ins	+A	FS, early stop					1			1	Scot_IDv1_y^4^
564,051	*prsA*	Foldase	ins	+T	FS, early stop							1	1	Unit_IDv1_z^1^
564,059	*prsA*	Foldase	del	−139 bp	FS, early stop							1	1	Unit_IDv1_ab^4^
564,128	*prsA*	Foldase	del	-A	FS, early stop				1		3		4	Heat_IDv1_a^4^, Heat_IDv1_b^4^, Heat_IDv1_y^1^, Will_IDv1_z^1^
1,075,265	Hypothetical	Hypothetical	non-syn	A->G	Asn->Asp		1						1	Xeni_IDv1_z^1^
1,285,883	*gerE*	Major transcriptional regulator of sporulation	ins	Phage integration		3								Xeni_IDv2_x^1^, Xeni_IDv2_y^1^, Xeni_IDv2_aa^4^
1,360,187	*lepA*	Translation elongation factor	ins	Phage integration				3						Fern_IDv1_yb^4^, Fern_IDv1_z^1^, Fern_IDv1_x^1^
1,366,666	*dnaJ*	Chaperone protein	del	−51 bp	del 17 aa		2		2				4	Will_IDv1_a^4^, Will_IDv1_x^1^, Xeni_IDv1_Ba^4^, Xeni_IDv1_Bb^4^
1,676,262	*bglG*	Mannitol operon activator	non-syn	T->A	Phe->Ile			1					1	Fern_IDv1_yb^4^
1,796,378	*rpiR*, *mutT*, *mdtJ*, *emrE* and *acrR*	Tagatose utilization, multi-efflux pump, ethidium bromide resistance, transcription	del	−2038 bp	4–5 gene deletion	2							2	Xeni_IDv2_x^1^, Xeni_IDv2_y^1^
2,034,618	Mobile element protein	Mobile element protein	non-syn	G->A	Ala->Thr	1			1		2	2	6	Heat_IDv1_x^1^, Unit_IDv1_o^3^, Xeni_IDv2_x^1^, Will_IDv1_x^1^
2,131,639	*cwlJ*	Spore cortex-lytic enzyme	syn	C->T	na							1	1	Unit_IDv1_ab^4^
2,187,569	*gntR*	Transcriptional regulator	del	-G	FS, early stop							1	1	Unit_IDv1_ab^4^
2,269,951	Mobile element protein	Mobile element protein	del	-C	FS, early stop		3	2		1	2	3	11	Fern_IDv1_z^1^, Fern_IDv1_x^1^, Heat_IDv1_z^1^, Scot_IDv1_r^2^, Unit_IDv1_m^3^, Unit_IDv1_o^3^, Xeni_IDv1_Ba^4^, Xeni_IDv1_Bb^4^
2,534,497	*ylzA*		non-syn	C->G	Arg->Pro		1						1	Xeni_IDv1_z^1^
2,703,591	*sigK*	RNA polymerase sporulation-specific sigma factor	SV		Disruption of the coding region			1						Fern_IDv1_z^1^
3,287,419	Intergenic		ins	+A	Unknown		1							Xeni_IDv1_z^1^

46Eight samples had mutations in the *prsA* gene, which encodes for the foldase protein PrsA. For seven of these samples, this was the sole mutation found in the genome. This membrane-bound lipoprotein assists in the folding of secreted proteins [47]. The surface-exposed region of this protein would be accessible as a phage receptor. Five unique mutations were observed in this gene: one small deletion, one deletion of 138 bp, two single-base insertions and a structural variant. These indels caused frameshifts in *prsA*. The structural variant was a large inversion between genome positions 339,868 (in a coding region annotated as hypothetical protein/transposase) and 563,806 (within the *prsA* coding region). One isolate with a *prsA* mutation had other mutations present in its genome (impacting *cwlJ* and *gntR*). Neither of these two mutations was observed in any other isolates.

Four isolates had a mutation in *dnaJ*, which encodes for a co-chaperone protein that increases the activity of the heat shock protein DnaK (Hsp70) [48]. This protein complex is essential for protein folding and is required for the replication of λ phage in *Escherichia coli* [49]. These four isolates all had the same 51 bp deletion near the N-terminal of the *dnaK* gene. This in-frame deletion resulted in the removal of 17 amino acids.

Seven samples had an intergenic mutation (genome position 303,314) in a *N*-acetylglucosamine (GlcNAc) biosynthesis gene cluster, just downstream of a predicted transcriptional regulator. GlcNAc is a component of the peptidoglycan layer. The genes in this cluster are essential in peptidoglycan synthesis and recycling. Isolate Fern_IDv1_yb had a nonsynonymous mutation in a mannitol operon activator, BglG family CDS. Isolate Xeni_IDv1_z had a nonsynonymous mutation in gene *ylzA*, which is a regulator of extracellular matrix formation in *Bacillus subtilis*. This ylzA mutation was accompanied by mutations in a hypothetical protein and an intergenic region. One Unit_IDv1-resistant isolate contained a mutation in *cwlJ*, a gene encoding a spore cortex-lytic enzyme. This enzyme is involved in peptidoglycan remodelling during spore formation. Two other mutations related to spore formation were observed. One inversion interrupted *sigK*, a sporulation-specific sigma factor, and the integration of phage Xeni_IDv2 into *gerE*, a transcriptional regulator of sporulation (this is discussed in a separate section below).

Two isolates that were resistant to phage Xeni_IDv2 contained a large deletion of five genes (a MurR/RpiR-family regulator, a mutT/nudix family protein, *emrE*, an *acrR* family regulator and a hypothetical protein). MurR/RpiR is a transcriptional regulator of sugar metabolism, including MurNAc synthesis. MutT/nudix family proteins are hydrolases with broad functions. *P. larvae* has four genes annotated as mutT/nudix family CDS. EmrE is likely a transporter that can provide resistance to ethidium bromide and methyl viologen. The *acrR* family gene is likely a transcriptional regulator of efflux pump proteins, possibly involved in biofilm signalling and formation. The MurNAc regulation, which may impact peptidoglycan structure, is unclear how these mutations would provide resistance to bacteriophages.

### Phage Fern_IDv1 and Xeni_IDv2 showed integration events in 3650 phage-resistant isolates

Apparent phage integration events were detected in all the Fern_IDv1- and Xeni_IDv2-resistant isolates. Evidence for this was first noticed in the breseq (see ‘Methods’) output as new junctions between the B-3650 and either Fern_IDv1 or Xeni_IDv2 phage genomes when breseq was run on a multifasta file containing both genomes. Subsequently, we undertook a careful investigation of potential recombination events by remapping reads and analysing read coverage. We found elevated (near host depth) coverage for Fern_IDv1 and Xeni_IDv2 genomes only in isolates challenged with Fern_IDv1 or Xeni_IDv2. Reads mapping to these phage reference genomes matched at 100% identity, suggesting that they were not misaligned. There are regions in B-3650 with high identity to Fern_IDv1 and Xeni_IDv2. However, in both cases, large regions of the genome with dissimilarity make it possible to distinguish the phage from the temperate phage regions in B-3650. Using the breseq junction coordinates and read mapping coordinates for soft-clipped reads aligned to the junctions, we identified the integration sites. For Fern_IDv1-challenged isolates, the Fern_IDv1 genome is inserted in B-3650 between bases 1,360,187 and 1,360,222, in the coding sequence for translation elongation factor LepA. These 35 bases are identical to a 35-base region in Fern_IDv1 at genome coordinates 19,500–19,535. This 35-bp region is now duplicated, with copies flanking the now-integrated Fern_IDv1 genome. The Fern_IDv1 genome is annotated with a 68 amino acid hypothetical protein in this location, which is directly 5′ to Fern_IDv1’s integrase gene. The Xeni_IDv2-resistant isolates now have a phage integrated between genome positions 1,285,883 and 1,285,893. These nine bases are now duplicated, flanking the now-integrated Xeni_IDv2 genome, which was disrupted at base 38,418. Integration occurred in the gene for the major transcriptional regulator of spore coat formation protein GerE in B-3650 and an intergenic region in Xeni_IDv2. We aligned reads to these new sequences, and in both cases, read coverage is even with no breaks, suggesting that these new junctions are correct. No breaks in coverage or soft-clipped reads were found in any isolates other than those challenged with Fern_IDv1 and Xeni_IDv2.

### Evolution usually results in broad phage resistance

To understand how the evolution of resistance will impact treatment with phage cocktails, we characterized the susceptibility of the 26 phage-resistant bacterial isolates to all 7 phages used in this study. In all cases, resistance to one phage resulted in cross-resistance to at least one other, typically closely related, phage (Fig. 2, Table S2). This generalization is especially true within the *Halcyoneviruses*, which are more closely related than the *Fernviruses* are. For example, isolates resistant to Heat_IDv1, Scot_IDv1 or Unit_IDv1 (*Halcyoneviruses*) almost always (14/15 isolates) showed some resistance to the other two phages in the cluster. One exception – an isolate that evolved resistance to Scot_IDv1 – was completely susceptible to Unit_IDv1. More variation in cross-resistance patterns occurred in hosts that evolved resistance to *Fernviruses* (Fern_IDv1, Will_IDv1, Xeni_IDv1 and Xeni_IDv2) [46]. Phages Fern_IDv1 and Will_IDv1, which have 99% ANI, usually infected the same host isolates, with two exceptions. Will_IDv1 could infect Xeni_IDv1_isolate_z and Heat_IDv1_isolate_a, while Fern_IDv1 could not. These two phage-resistant isolates do not share any mutations. The most distantly related phage in the Fern cluster (Xeni_IDv1, ~80% ANI) could usually (5/6 phage-resistant isolates tested) infect Fern_IDv1 and Will_IDv1 resistant hosts. The opposite was not true; Xeni_IDv1 challenged isolates were usually resistant to Fern_IDv1 and Will_IDv1. Xeni_IDv1 also differed from Fern_IDv1 and Will_IDv1 in its ability to infect other phage-resistant isolates. For example, Xeni_IDv1-resistant hosts were resistant to Xeni_IDv2, but not Will_IDv1 and Fern_IDv1. While the general pattern is that resistance evolution includes closely related phages, there were many sporadic instances of cross-resistance to more distantly related phages. For example, Heat_IDv1-resistant isolate b was resistant to closely related phages Scot_IDv1 and Unit_IDv1 as expected, but also to phages Fern_IDv1 and Will_IDv1, which are in a different genus with <30% sequence identity. Xeni_IDv2_isolate_Aa gained broad resistance to many of the phages. Interestingly, all six Xeni-resistant isolates were resistant to Heat_IDv1, which has low nucleotide sequence identity to Xeni phages.

In some instances, resistance was not complete, and occasional plaques were observed on spot and phage overlay plates. To test if resistance was stronger against the challenge phage than against phages that the host acquires cross-resistance to, we compared the EOP of all phages able to grow on a partially resistant host. EOP is a commonly used phenotype that is calculated by measuring the number of viruses that can form plaques. We found a difference in EOP between these two groups (post hoc Tukey HSD (Honestly Significant Difference) test, *P*=2E−4). This result can be visualized in Fig. 2 by comparing the transparency of dots along a row to the dot in the box. The transparency of dots in the box is slightly less transparent than dots outside the box. This effect is small, with the average EOP of a phage on a partially resistant host againts which it was challenged being 1.6 times lower than other phages that it gained cross-resistance towards.

### Growth defects in resistant isolates

**Fig. 4.**
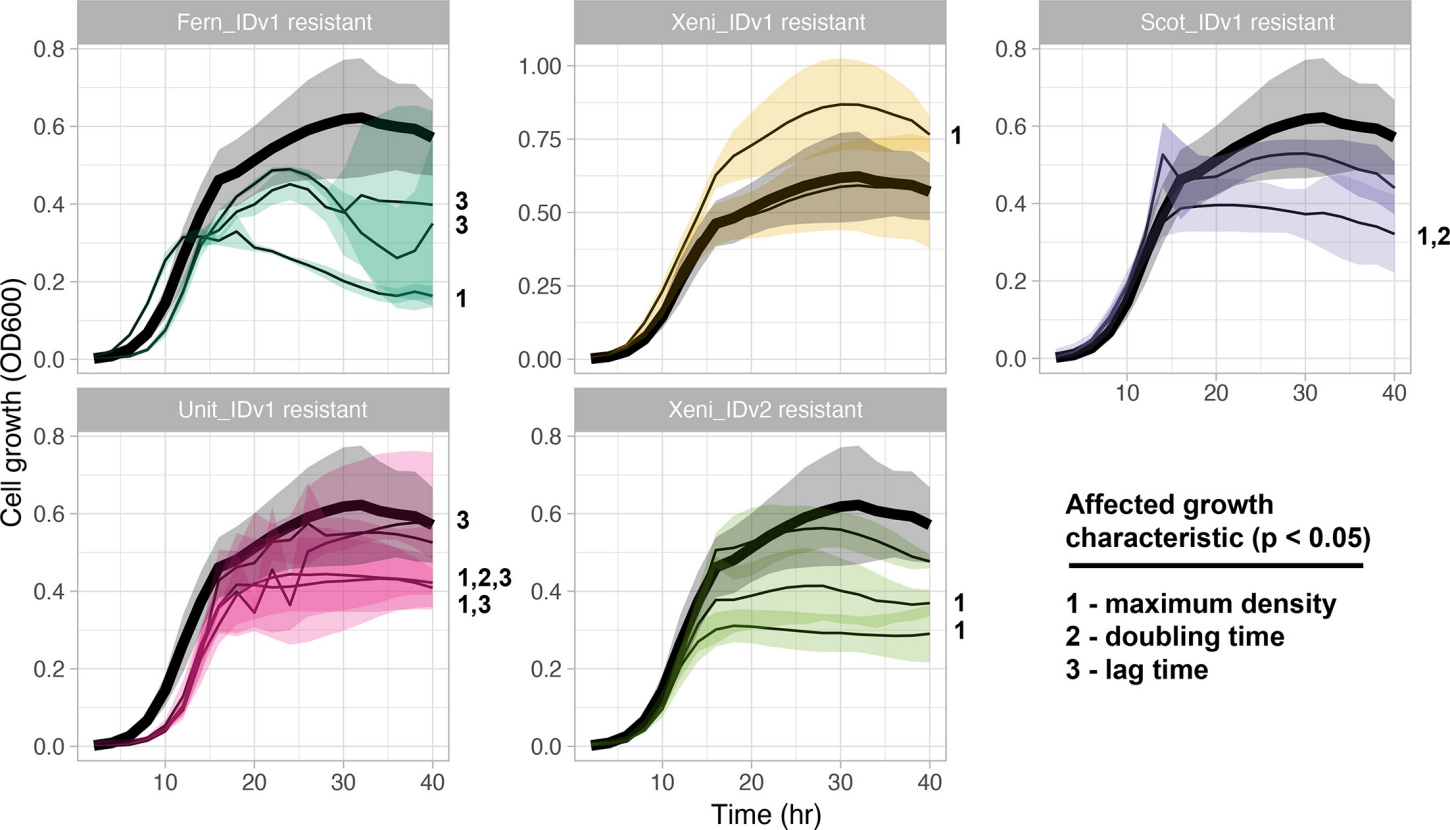
Phage-resistant variants have growth defects. Growth curves of phage-resistant isolates (thin black lines) compared to parental strain B-3650 (thick black line). The standard deviation for at least three replicates is shown by the shaded region. Growth characteristics extracted from growth curves using the gcplyr R package (see ‘Methods’).

We compared the growth curves of 14 of the phage-resistant isolates of *P. larvae* to the parental B-3650 strain and found that most (10/14) of the isolates had growth defects when grown without phages (Fig. 4, Tables S3 and S4). Lag time, doubling time and maximum density were calculated and used as response variables in linear models with either phage or bacterial isolate as predictor variables. Both predictor variables showed significant global effects (*P*=3.1E−4 for phage, *P*=3.5E−5 for isolate, *F*-test in R using summary function). Post hoc tests on individual isolates indicated that all phages except for Xeni_IDv1 caused a significant reduction in maximum density in at least one of the resulting phage-resistant isolates (*P*<0.05, df=48, t-test). Maximum density was the most impacted dependent variable, differing from B-3650 for 7 of the 14 isolates tested (*P*-values and coefficients in Tables S3 and S4). One isolate (Xenia-resistant isolate Bb) had an increase in maximum density compared to B-3650 (*P*=0.002, t-test). All other significantly different isolates (Fern_IDv1-resistant isolate yb, Scot_IDv1-resistant isolate b, Unit_IDv1-resistant isolate y, Unit_IDv1-resistant isolate z, Xeni_IDv2-resistant isolate x and Xeni_IDv2-resistant isolate y) had reduced maximum growth density. Thus, although we observe growth effects of phage resistance in many isolates, there is variation even within each exposure group (one challenge phage type).

### Phage variants can infect resistant hosts

**Fig. 5.**
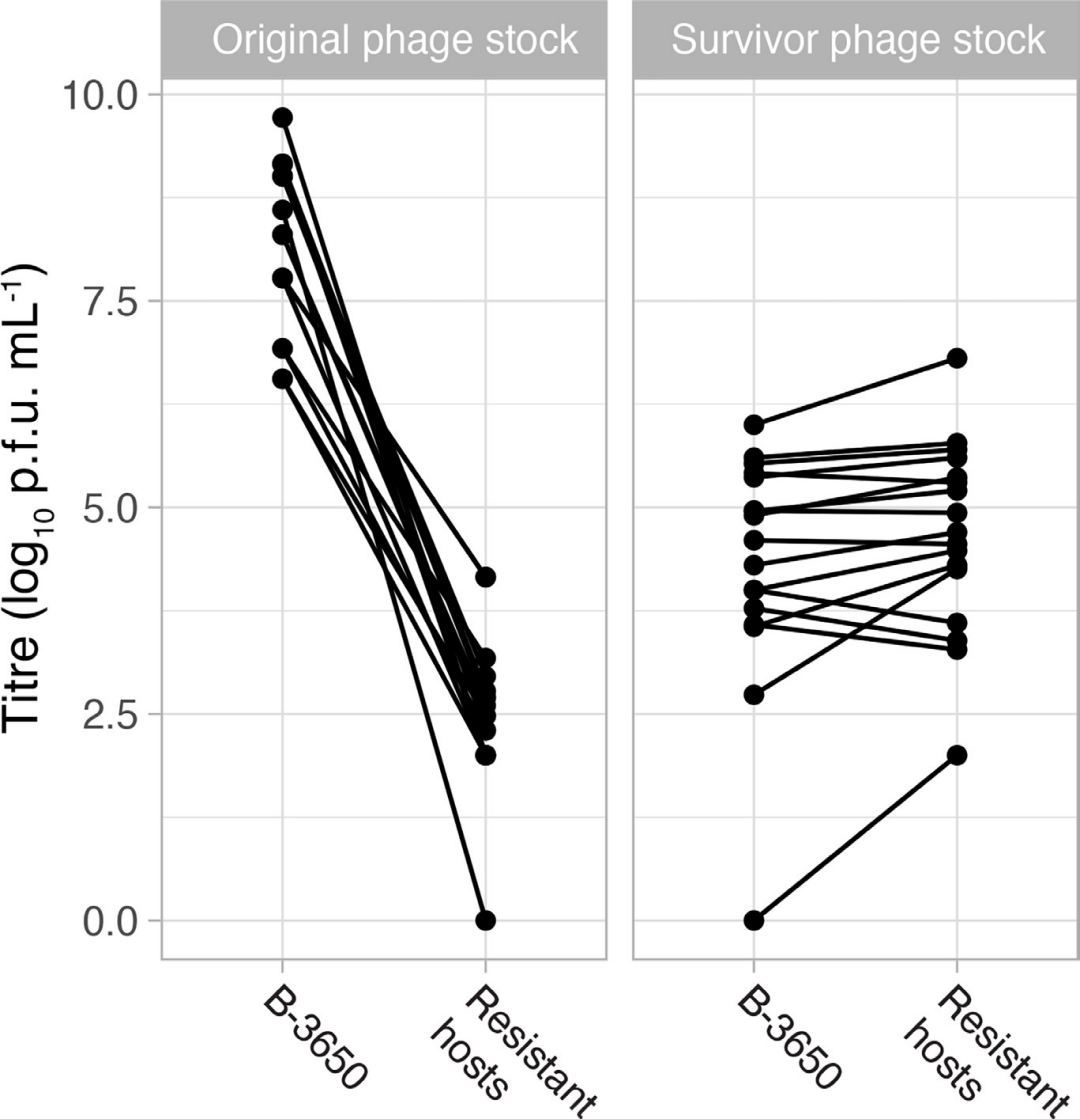
The efficiency of plaquing assays revealed that a small subset of the phages could overcome resistant bacteria. A fraction (18/28) of the phages could form plaques on resistant hosts with very reduced plating efficiency (left panel). When cultivated on resistant hosts, these ‘survivor’ phage isolates had higher plating efficiency on the resistant hosts than the parental host (right panel).

While verifying the resistance of phage-resistant isolates, we discovered plaques on 18 of the 26 phage-resistant isolates, suggesting that either resistance is partial or a portion of the phage population can overcome resistance. We further investigated this by measuring the relative EOP of these phages on B-3650 and phage-resistant isolates (Fig. 5, Table S5). In our case, we compared how many plaques formed on the ancestor versus the resistant hosts. The mean EOP of the initial phage lysates on resistant hosts was 4.5E−5 ± 2.0E−5 (±1 se). We were unsure if the plaques on resistant hosts were genetic variants with the ability to plaque on resistant hosts or if resistance was incomplete, allowing some phage growth. To investigate this, we picked one plaque off every phage-resistant strain and measured the EOP of these picked plaques (titre on resistant host/titre on B-3650). The mean EOP of these plaques was 9.6±2.0 (±1 se). The minimum EOP for these ‘survivor’ phages was 0.4, meaning that just under half of the phages could plaque on their resistant host after just one round of selection. The EOP of this phage changed from 6.9E−8 before this round of selection to 0.4 after, meaning that only about 1 in every 100,000,000 phages from the original phage population formed plaques on the original host, while 4 out of 10 could after 1 round of selection. Therefore, we conclude that rare genetic variants likely exist in the original phage stock that facilitate growth on evolved hosts. These ‘survivor’ phages grew slightly worse (lower litres) on the parental host (Fig. 5, *P*=0.03, df=15, t-test), showing that there is a trade-off associated with growth on phage-resistant hosts.

### Reduced virulence in bee larvae

**Fig. 6.**
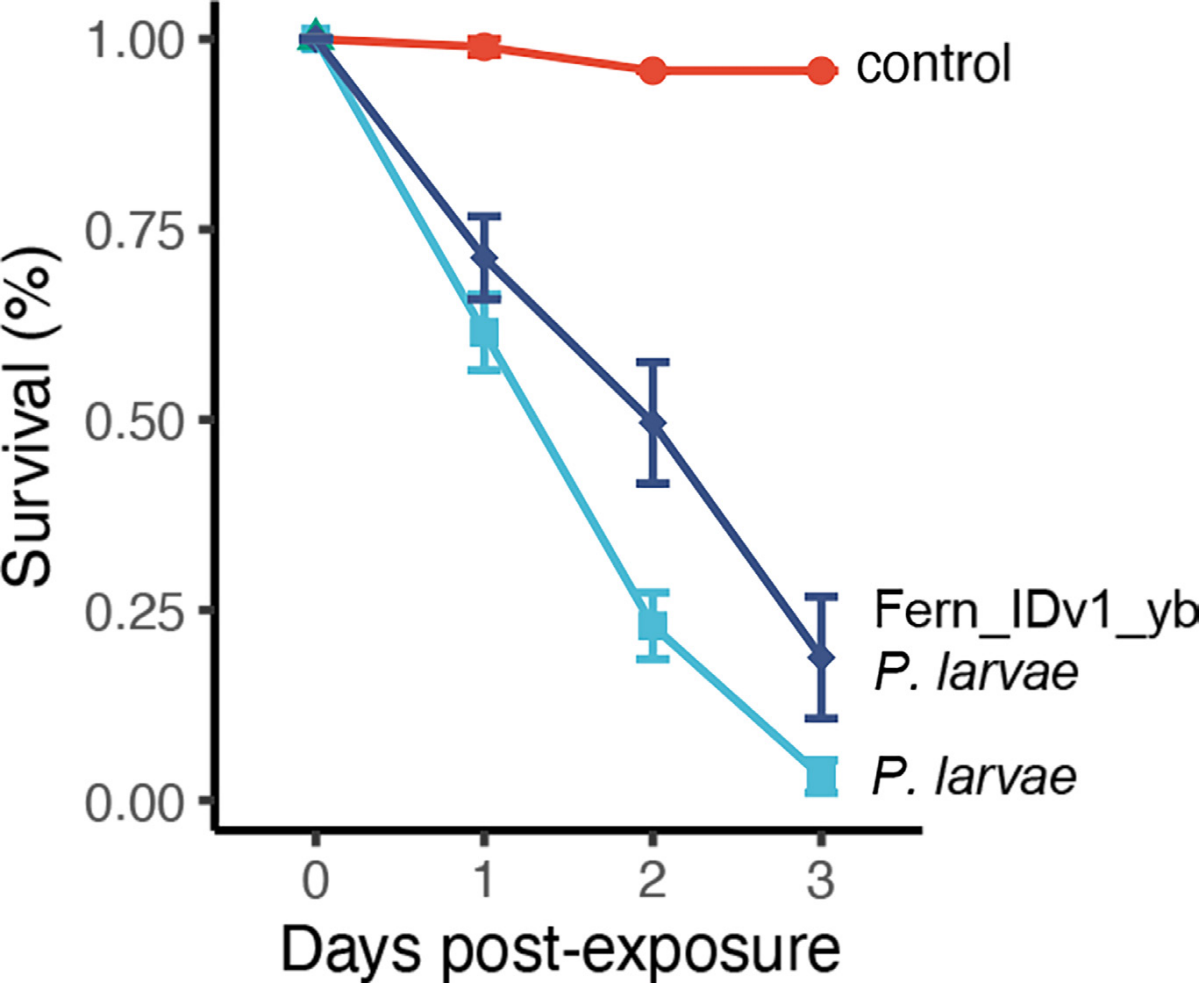
A phage-resistant *P. larvae* isolate is less virulent than its ancestor *P. larvae*. Recently, hatched larvae were reared with diets containing *P. larvae* B-3650, Fern_IDv1-resistant *P. larvae* B-3650 or media only. Twelve, 16 or 48 larvae were used in replicate experiments. Standard error bars are shown.

To determine if the growth defects caused by phage resistance are relevant to bees, we performed a small larval infection trial with four treatments. Newly hatched larvae were grafted into queen cell cups fed standard feed (see ‘Methods’) or standard feed with the addition of phage Fern_IDv1+*P. larvae* strain B-3650, *P. larvae* strain B-3650 alone or Fern_IDv1-resistant * P. larvae* strain B-3650 (isolate yB). This mutant has one non-synonymous mutation (F109I) in a mannitol operon activator gene and is a lysogen. Nearly all (69/72) the mock-infected larvae survived (Fig. 6). Larvae infected with *P. larvae* and treated with Fern_IDv1 phage survived at ~75%, which is less than the control (*P*=5E−5, log-rank test with BH correction, *n*=1248, df=3). Larvae infected with *P. larvae* strain B-3650 had the worst survival, with only 3% of them surviving 3 days post-infection. Eighteen per cent of the larvae infected with Fern_IDv1-resistant strain B-3650 survived, an 83% increase in survival compared to WT B-3650 (*P*=0.001, log-rank test with BH correction, *n*=1248, df=3).

## Discussion

Using bacteriophages to treat bacterial infections of honey bees is a promising alternative to antibiotic use, but the propensity of these pathogens to become phage resistant has not yet been assessed. We sequenced *P. larvae* isolates that evolved resistance to seven of its associated phages and characterized resulting phenotypic changes in the host. This work shows that these bacteria can become resistant when challenged with single phages, provides insight into likely phage receptors and shows how bacterial growth is impacted by phage resistance mutations.

Phage resistance readily arose in an ERIC-I strain of *P. larvae*, a group of pathogens commonly observed in honey bee colonies [31]. Even though *P. larvae* has several anti-phage systems [50], it is susceptible to many previously isolated phages. To ensure that the defence systems in the *P. larvae* isolate we are working with are the same as what was previously characterized, we sequenced the genome of our isolate and ran DefenseFinder [51]. We observed no changes in any phage defence-related genes and no incorporation of new CRISPR protospacers. When we challenged *P. larvae* with seven different phages, resistant isolates were observable within 2 days. This type of experimental evolution has been done with many phage-host combinations and is commonly used to identify potential phage receptors [52,56]. Each of the 26 resistant isolates that were sequenced had between one and three mutations compared to the ancestor. Other phage challenge evolution experiments have found multiple mutations in resistant hosts [55,56], and although there are no empirical measurements of mutation rate for *P. larvae*, it is not unexpected to observe multiple mutations based on mutation rates measured for other *Paenibacillus* species. In the 26 phage-resistant isolates, only 19 unique mutations were found. Many (10/19) of these mutations were indels, one being a large deletion of five genes. The annotation of many of the affected genes suggests that the mutations have some plausible role in phage receptor presentation; either the genes encode surface proteins or they are involved in sugar or peptidoglycan biosynthesis. It is worth mentioning that all *P. larvae* phage genomes analysed in a recent study of 48 phages contained a *N*-acetylmuramoyl-l-alanine amidase [24]. These endolysins are likely involved in the lysis of host cells. Thus, is it conceivable that the peptidoglycan mutations we observed somehow alter the cell wall structure, preventing lysis rather than entry.

There was some phylogenetic specificity of resistance mutations. Hosts’ resistance to phages in the *Fernviruses* (Fern_IDv1, Will_IDv1, Xeni_IDv1 and Xeni_IDv2) had mutations impacting *dnaJ*, a mannitol operon activator and a set of three mutations (hypo, ylzA and intergenic). One isolate (Will_IDv1-resistant isolate z) had a mutation in *prsA*. The cluster containing Heat_IDv1, Scot_IDv1 and Unit_IDv1 was impacted by five different mutations impacting *prsA*. Isolates without *prsA* mutations had a mutation in a transcriptional regulator of *N*-acetylgalactosamine. It is unclear if the impact of this mutation affects metabolism or cell surface sugar molecule presentation because the genes under the control of this regulator region could impact both [57,58]. The conversion of B-3650 into lysogens by Fern_IDv1 and Xeni_IDv2 provided resistance against these phages and provides support for the careful use of temperate phages in phage therapy. These phages would be poor choices for treating *P. larvae* infections in honey bee colonies. It is worth noting that B-3650 carries prophages with regions of high sequence identity to Fern_IDv1 and Xeni_IDv2; thus, it may be advisable to avoid using phages that share sequence homology to prophages in a pathogen’s genome. Four of the six Fern_IDv1 and Xeni_IDv2 phage-resistant isolates had additional mutations in their genomes. It is unclear how these mutations contribute (if at all) to resistance.

Phage cross-resistance was largely clustered by phylogenetic relatedness of the challenge phages. Evolving resistance to one phage often confers resistance to phages in the same phylogenetic cluster. Some interesting exceptions to this rule are not easily reconciled with the dogma that phages are generally highly host-specific. Hosts that evolved resistance to Xeni_IDv2 acquired broad protection from phylogenetically diverse phages. These isolates are all lysogens, but isolates *x* and *y* have additional mutations, including a large deletion of five genes (both isolates) and a non-synonymous mutation in a mobile element protein (isolate *x*). Others have observed lysogeny providing protection to distantly related phages; however, the mechanism of protection was not resolved [59]. Isolates that evolved resistance to Will_IDv1 and Xeni_IDv1 were also commonly resistant to Xeni_IDv2. No resistance mutations were found in common between Xeni_IDv2 and Xeni_IDv1/Will_IDv1 resistant hosts, so it is unclear how cross-resistance against Xeni_IDv2 is gained. Will_IDv1 resistance mutations involve genes *dnaJ* and *prsA*. Given that cross-resistance to distantly related phages is rare in our results and studies performed by different groups, utilizing phage cocktails made of distantly related phages is likely to decrease the chance of cross-resistance evolution.

Several instances of asymmetric resistance acquisition were observed. For example, isolates that gained resistance to Will_IDv1 were not resistant to Xeni_IDv1, but those resistant to Xeni_IDv1 were resistant to Will_IDv1. Isolates resistant to Xeni_IDv2 were not resistant to Will_IDv1, but Will_IDv1-resistant isolates were resistant to Xeni_IDv2. The Scot_IDv1/Heat_IDv1/Unit_IDv1-resistant isolates were not resistant to Xeni_IDv1, but Xeni_IDv1-resistant isolates gained partial resistance to these three phages. Others have also reported asymmetric cross-resistance. Among 263 phage-resistant isolates of *P. aeruginosa*, Wright *et al.* [60] found isolates to be cross-resistant to 10–80% of the other 27 phages used in the study [60]. Cross-resistance is also common when phage receptors are transporters involved in antibiotic resistance [61,63]. Gao *et al.* [64] reported both asymmetric cross-resistance and somewhat sporadic patterns of cross-resistance in *Salmonella enterica* [64]. We found more effective resistance to the phage that was used during evolution than cross-resistant phages, as evidenced by lower EOP values for challenge phages than cross-resistant phages (post hoc Tukey HSD test, *P*=2E−4). Our results are consistent with results from others showing occasional resistance against phages that are distantly related to the challenge phage [60,65, 66]. This suggests that even when combinations of phylogenetically divergent phages are used to treat bacterial infections, there is a chance that universal resistance will evolve. Sequencing the genomes of resistant isolates may provide some predictions on the breadth of cross-resistance. Wright *et al.* [60] propose that mutations in potential cell-surface receptors (e.g. LPS) are less likely to confer broad resistance than resistance mutations that change regulator genes (e.g. transcriptional regulators).

In this study, we found evidence that phage resistance can come at a cost in terms of growth rate and pathogenicity. However, like other studies have found [67,68], these costs are not universal – we observed growth defects in culture for half of the isolates that we tested. The one isolate that we tested in bee larvae was less virulent than B-3650, but more isolates should be tested, particularly those that do not have reduced maximum density in culture. Many other studies have found that evolution to resist phage infection is accompanied by fitness costs, particularly in the context of a natural microbial community (reviewed in [69]), and thus, phage therapy is unlikely to result in widespread resistance against phages [70]. Phages that have been preadapted (e.g. ‘trained’) to overcome bacterial resistance have been shown to reduce the emergence and rise of phage-resistant genotypes [71,72]. In our experiments, we observed phages that overcame resistance in 18 of the 26 phage challenges. These phages plaqued on phage-resistant isolates but grew to lower litres on the starting, non-resistant host (Fig. 5). Plaquing efficiency assays revealed that a small subset of the phages could overcome resistant bacteria. A fraction (18/28) of the phages could form plaques on resistant hosts with very reduced plating efficiency (Fig. 5, left panel). When cultivated on resistant hosts, these ‘survivor’ phage isolates had higher plating efficiency on the resistant hosts than the parental host (Fig. 5, right panel). We interpret this reduction in titre as a trade-off for being able to utilize the phage-resistant hosts. The number of survivor phages observed is consistent with a hypothesis that these phages overcome resistance via mutation, although there are other potential explanations. Given a dsDNA viral mutation rate of ~1×10^−7^ mutations per nucleotide per infection cycle [43] and roughly 35–45 kb genome sizes and the 10^7^ phages plated, we calculate roughly 40,000 mutants per plate. This number is much larger than the number of plaques we observed on the lawns of phage-resistant isolates. Coevolution between phages and their hosts is common in many systems [69,73, 74], and although we did not sequence our survivor phages, coevolution between * P. larvae* phages and resistant *P. larvae* isolates is certainly possible based on the presence of these phage isolates.

## Abbreviations

AFB: American Foulbrood
ANI: average nucleotide identity
BHI: brain heart infusion
EOP: efficiency of plating
ERIC: enterobacterial repetitive intergenic consensus
MOI: multiplicity of infection
